# Metabolic syndrome among Tunisian psychiatric patients receiving antipsychotics: Prevalence and associated factors

**DOI:** 10.1192/j.eurpsy.2026.11789

**Published:** 2026-07-31

**Authors:** Y. Ben Othmene, A. Aissa, S. Aouadi, M. Gardabou, R. Hosni, A. Ouertani, R. Jomli

**Affiliations:** 1 Avicenne psychiatry department; 2 Outpatient department, Razi Hospital, Tunis, Tunisia

## Abstract

**Introduction:**

Psychiatric patients have a higher mortality rate than the general population, predominantly from cardiovascular disease associated with metabolic syndrome (MetS).

This problem arises from combined genetic, biological, behavioral and iatrogenic factors.

**Objectives:**

This study aimed to determine the prevalence of MetS among Tunisian psychiatric patients treated with antipsychotics and its associated factors.

**Methods:**

A cross-sectional descriptive and analytical study, conducted between January and June 2025, involving Tunisian psychiatric patients at the Avicenne Psychiatry Department of Razi Hospital. Data were collected using a file and two Arabic-translated questionnaires:

The International Physical Activity Questionnaire (IPAQ): categorizing physical activity into: Inactive, Minimally active and HEPA-active.

The Mediterranean Diet Adherence Screener (MEDAS): with two categories: moderate to weak adherence ≤7; good to very good adherence ≥8.

MetS was defined according to NCEP ATP III criteria.

**Results:**

Fifty-two patients were included with 71% being male. Their mean age was 38 [19-62] years. Overall, 69% were single, 23% married and 8% separated. Most participants (79%) had not completed secondary education and 63% were professionally inactive. Smoking was prevalent among 75% of the participants while 8% reported alcohol consumption.

The majority (77%) had no underlying medical conditions. Psychiatric diagnoses included schizophrenia (42%), bipolar I disorder (35%), bipolar II disorder (4%), schizoaffective disorder (13%) and other psychiatric conditions. Most patients (73%) were on atypical antipsychotics and 17% received concurrent typical and atypical agents. Among those on atypical antipsychotics, 62% were prescribed olanzapine or clozapine.

Good adherence to the Mediterranean diet was observed in 40% of participants.

Regarding physical activity, 38% were inactive, 31% minimally active and 31% HEPA-active.

Thirty–eight percent met the criteria for MetS.

A higher body-mass index (BMI) was significantly associated with the presence of MetS (p=0.014).

Longer exposure to antipsychotic medication was also associated with MetS but this was not statistically significant when adjusted for BMI.

No protective factors were identified.

**Conclusions:**

This study showed that higher body-mass index was significantly associated with MetS, while neither Mediterranean diet nor physical activity showed a protective association.

These findings highlight the importance of careful monitoring of antipsychotic treatment and the implementation of weight management strategies in psychiatric care.

**Disclosure of Interest:**

None Declared

